# Exclusion of patients living with HIV from cancer immune checkpoint inhibitor trials

**DOI:** 10.1038/s41598-021-86081-w

**Published:** 2021-03-23

**Authors:** Kruti B. Vora, Biagio Ricciuti, Mark M. Awad

**Affiliations:** 1 Harvard Medical School, Boston, MA USA; 2 Lowe Center for Thoracic Oncology, Dana-Farber Cancer Institute, Harvard Medical School, 450 Brookline Avenue, Boston, MA 02215 USA

**Keywords:** Health care, Oncology

## Abstract

Emerging retrospective and prospective studies indicate that immune checkpoint inhibitors (ICIs) can be safe and effective cancer treatments among people living with human immunodeficiency virus (PLWH), however this high-cancer-risk population has often been excluded from groundbreaking cancer ICI trials. Our study aimed to characterize the current rate of exclusion and conditional inclusion of PLWH in cancer ICI trials by tumor type, trial phase, and year. ClinicalTrials.gov cancer ICI trials with planned starts between 1/1/2019 and 10/20/2020 were identified. Based on trial eligibility criteria, trials were categorized as “excluded” if PLWH could not enroll, “conditionally included” if only PLWH with adequate immune function were allowed, or “included/not specified” if HIV was not mentioned in the eligibility criteria. Trials from 2014 were separately collected for comparison over time. The number of trials excluding PLWH were compared to the included/not specified group using Fisher’s exact test. Of 809 trials analyzed from 2019 to 2020, 74.4% excluded, 6.9% conditionally included, and 18.7% included/did not specify PLWH. Early phase trials excluded PLWH more frequently than late phase trials. The 2019–2020 trial cohort showed no significant change in exclusion of PLWH compared to 2014. Despite increasing evidence for safe and effective ICI use for PLWH, most cancer ICI trials exclude PLWH and few studies permit PLWH to participate, even if HIV is well-controlled.

## Introduction

The human immunodeficiency virus (HIV) epidemic affected millions worldwide and caused outright discrimination against those who were infected with HIV, acquired immunodeficiency syndrome (AIDS) or perceived to be linked to HIV due to their sexual orientation, gender, or identity^1,2^. In 2019, 38 million people around the world were living with HIV with 1.7 million new cases diagnosed that year^1^. While HIV continues to affect millions each year, the development of antiretroviral therapy (ART) has revolutionized the treatment of this illness, making long-term survival and chronic management possible. Unfortunately, people living with HIV (PLWH) continue to be marginalized in many communities around the world. In medicine, PLWH often do not receive the same level of care as their non-HIV-infected counterparts, even if their illness has been well-controlled with ART and they have normal CD4^+^ T cell counts. One in eight PLWH from data gathered across 50 countries in the People Living with HIV Stigma Index reported being denied health services due to the stigma of their disease^2,3^.

One example of inadequate access to care for PLWH is the exclusion of this population from potentially life-extending clinical trials. This was the case for several groundbreaking immune checkpoint inhibitor (ICI) cancer trials despite the fact that PLWH are at increased risk of developing and dying from cancer, both AIDS-defining and non-AIDS-defining^4–9^. PLWH are 500 times more likely to develop Kaposi sarcoma, 12 times more likely to be diagnosed with non-Hodgkin’s lymphoma and three times higher risk for cervical cancer^6,7,10^. Infection with HIV also places these patients at a higher risk of developing non-AIDS-defining cancers such as cutaneous squamous cell carcinoma, Merkel-cell carcinoma, and conjunctival squamous cell carcinoma^11^. With improvements in ART efficacy, the population of PLWH has aged, thus increasing the risk of PLWH developing cancers that affect non-HIV-infected aging populations^11^. PLWH are not only at higher risk of developing cancer, but they also have a higher cancer-specific mortality than their non-HIV-infected counterparts with cancer^8,9^. Despite the increased prevalence and aggressive nature of cancers for PLWH, PLWH have historically not had the same access to cancer treatments as their non-HIV-infected counterparts.

Emerging retrospective and prospective studies indicate that ICIs can be safe and effective cancer treatments among PLWH^4,12–14^. A recent review of ICI use in PLWH with advanced cancer found that objective response rates were 30% for non-small cell lung cancer, 27% for melanoma, and 63% for Kaposi sarcoma^4^. For 93% of patients with undetectable HIV viral loads, viral load remained suppressed. CD4^+^ counts increased by a mean of 12.4 cells/μL, and grade 3 + immunotherapy-related adverse events were noted in six out of 70 patients. Several other studies also found clinical benefit of immunotherapy for PLWH and cancer^12–14^.

With increasing evidence for the safety and efficacy of immunotherapy use in PLWH, the Food and Drug Administration (FDA) released a 2020 guidance statement advocating for the inclusion of PLWH with adequate immune function in cancer trials^15^. The guidance recommends that patients with CD4^+^ T-cell counts ≥ 350 cells/μL and without AIDS-defining opportunistic infections within the past 12 months be eligible for any cancer trial. For non-curative, advanced cancer treatments, trial participants should be on ART for four or more weeks with a viral load of below 400 copies/mL before trial enrollment. The guidance also details exceptions for PLWH who don’t meet these criteria. This FDA guidance was also supported by the American Society of Clinical Oncology (ASCO) and Friends of Cancer Research^16^. To better understand cancer treatment options for PLWH and modern-day clinical trial norms for cancer ICI trials, our study sought to characterize the rate of exclusion and conditional inclusion of PLWH in cancer ICI trials by tumor type, trial phase, and trial year.

## Results

**Figure 1:**
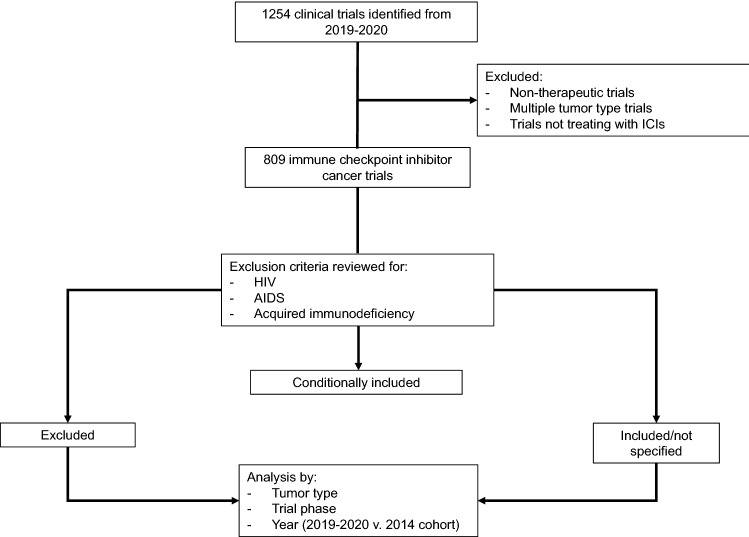
Cancer immune checkpoint inhibitor 2019–2020 trial cohort collection, categorization, and analysis methods. Cancer immune checkpoint inhibitor trials with planned start dates in 2019 and 2020 were collected using keyword searches, resulting in 1254 trials. Non-therapeutic trials, trials allowing multiple tumor types, and trials not treating with immune checkpoint inhibitors were excluded, leaving 809 remaining trials. Exclusion criteria from enrollment in these trials were reviewed for exclusion of human immunodeficiency virus, acquired immunodeficiency syndrome, or acquired immune deficiency to categorize trials as “excluded,” “conditionally included,” or “included/not specified” if the criteria did not contain these terms. The numbers of trials excluding patients living with HIV versus included/not specified trials were then compared by tumor type, trial phase and year. A separate 2014 cohort was used for comparison by year.

**Figure 2:**
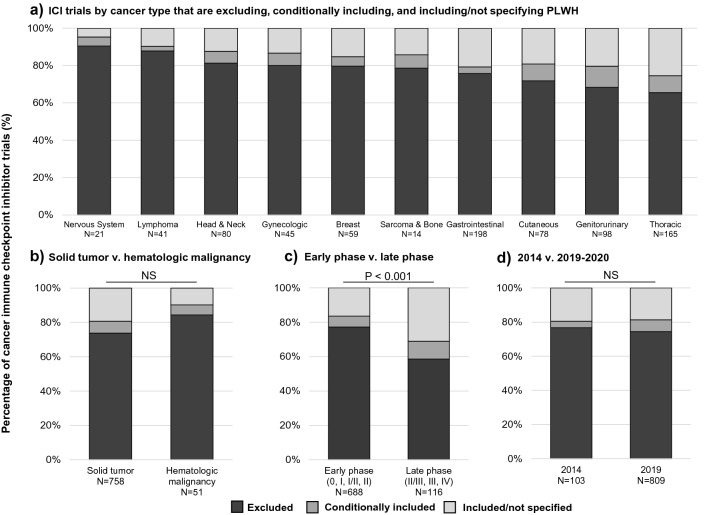
Percentage of cancer immune checkpoint inhibitor trials that excluded, conditionally included, and included/not specified PLWH. **(a)** Rate of 2019–2020 cancer immune checkpoint inhibitor trials excluding, conditionally including, and including/not specifying PLWH by grouped cancer type. Cancer types with fewer than 10 trials were excluded from this analysis. **(b)** Solid tumor versus hematologic malignancy 2019–2020 cancer immune checkpoint inhibitor trials that are excluding, conditionally including, and including/not specifying PLWH. **(c)** Early phase versus late phase 2019–2020 cancer immune checkpoint inhibitor trials that are excluding, conditionally including, and including/not specifying PLWH. Five trials that did not have trial phase listed on ClinicalTrials.gov were excluded from this analysis. **(d)** Cancer immune checkpoint inhibitor trials from 2014 versus 2019–2020 that are excluding, conditionally including, and including/not specifying PLWH. P-values shown reflect the comparison of trials excluding PLWH versus included/not specified trials by tumor type, phase, and trial year. *NS* not significant.

All ICI trials with were sorted into “excluded,” “conditionally included,” and “included/not specified” categories (Fig. 1). Of the 809 ICI trials analyzed with 2019–2020 start dates, 602 (74.4%) excluded PLWH, 56 (6.9%) trials conditionally included PLWH, and the remaining 151 (18.7%) trials belonged to the included/not specified category (Table 1). When trials were grouped by tumor type, the majority of trials across all tumor types excluded PLWH. These exclusion rates ranged from 65.5% for thoracic cancers to 90.5% for nervous system tumors (Fig. 2a). Conditional inclusion rates ranged from 2.4% (lymphoma) to 11.2% (genitourinary) by tumor type (Fig. 2a).

**Table 1:** Number and percentage of cancer immune checkpoint inhibitor trials excluding, conditionally including, or including/not specifying patients living with HIV.

Trial category	Excluded (%)	Conditionally included (%)	Included/not specified (%)
**Tumor type**			
Nervous system	19 (90.5%)	1 (4.8%)	1 (4.8%)
Lymphoma	36 (87.8%)	1 (2.4%)	4 (9.8%)
Head and neck	65 (81.3%)	5 (6.3%)	10 (12.5%)
Breast	47 (79.7%)	3 (5.1%)	9 (15.3%)
Sarcoma and bone	11 (78.6%)	1 (7.1%)	2 (14.3%)
Gastrointestinal	150 (75.8%)	7 (3.5%)	41 (20.7%)
Gynecologic	36 (80.0%)	3 (6.7%)	6 (13.3%)
Cutaneous	56 (71.8%)	7 (9.0%)	15 (19.2%)
Genitourinary	67 (68.4%)	11 (11.2%)	20 (20.4%)
Thoracic	108 (65.5%)	15 (9.1%)	42 (25.5%)
**Solid vs. hematologic malignancy**			
Solid	559 (73.7%)	53 (7.0%)	146 (19.3%)
Hematologic	43 (84.3%)	3 (5.9%)	5 (9.8%)
**Trial phase**			
Early phase	531 (77.2%)	44 (6.4%)	113 (16.4%)
Late phase	68 (58.6%)	12 (10.3%)	36 (31.0%)
**Trial year**			
2014 cohort	79 (76.7%)	4 (3.9%)	20 (19.4%)
2019–2020 cohort	602 (74.4%)	56 (6.9%)	151 (18.7%)

The number of trials excluding PLWH did not significantly differ between hematologic malignancy and solid tumor trials (P = 0.09, Fig. 2b). Early phase trials were more likely to exclude PLWH compared to late phase trials (P < 0.001, Fig. 2c). When compared to the 2014 ICI trial cohort, trials from 2019–2020 showed no significant difference in exclusion of PLWH (P = 1.00, Fig. 2d). Across the 912 trials analyzed in our study between 2014 and 2019–2020, none specifically listed PLWH as being included in the trial. The full categorization of trials from 2014 and 2019–2020 as “excluded,” “conditionally included,” or “included/not specified” can be found in Supplementary Appendix 1.

## Discussion

Despite increasing evidence for the safety and efficacy of ICIs for PLWH, 74.4% of recent cancer ICI trials excluded all PLWH regardless of patients’ viral loads, CD4^+^ T-cell counts, or HIV treatment courses, limiting treatment options for cancer patients with HIV. The minority of trials conditionally included PLWH even though the 2020 ASCO-backed FDA consensus guidelines recommended inclusion of PLWH with adequate immune function in cancer trials. Early phase trials were more likely to exclude PLWH than late phase trials, and since 2014, the relative distribution of exclusion in cancer ICI trials has continued to be high. To our knowledge, this is the first study characterizing the exclusion of PLWH in recent cancer trials. The data from this study highlights the continued treatment disparities for PLWH through lack of access to potentially life-extending cancer treatments.

The lack of consistent evidence-based guidelines in trial development for patients with HIV and cancer may have contributed to the exclusion of PLWH from cancer ICI trials. Most trials in this study did not list an explanation of why they chose to exclude PLWH from their trials. Those that did explain a reason for PLWH exclusion cited various potential risks due to marrow-suppressive agents, ICIs, other study drugs, or drug-drug interactions with ART. As one example, a trial stated, “The need to exclude patients with AIDS is based on the lack of information regarding the safety of nivolumab in patients with active HIV infection.” However, with emerging data suggesting that ICI use is safe and effective in PLWH, PLWH should be allowed to access these potentially beneficial therapies.

The 2020 FDA consensus guidelines on the inclusion of PLWH in cancer trials suggest new clinical trial norms that offer safe, equitable access to cancer treatment. The trials conditionally including PLWH in this study did not have inclusion cutoffs of CD4^+^ T-cell count, ART duration, or viral load that were consistent with the FDA 2020 guidance cutoffs. Trial norms must change to be evidence-based, safe, and equitable. Hopefully further research in this field can safely include larger studies of PLWH on ICI therapy to better understand the effects of ICI for patients with low CD4^+^ count or active HIV and the interactions between ART regimens and ICIs to further drive evidence-based trial criteria.

One limitation of this study was our reliance on the ClinicalTrials.gov exclusion criteria being accurate and up to date. We attempted to address this limitation by searching for the full study protocols of trials that did not mention PLWH in the ClinicalTrials.gov eligibility criteria. When full protocols were found through published manuscripts, posters, ClinicalTrials.gov study documents, and institutional trial databases, PLWH were often additionally excluded and never explicitly included in the trials. The remaining “included/not specified” trials had no additional eligibility criteria published, however, it is very possible that many “included/not specified” trials may actually exclude PLWH. Thus, the results of this investigation should be interpreted as the most conservative estimation of the exclusion of PLWH in cancer ICI trials.

Another limitation of this study was that it was restricted to cancer ICI trials. There are several alternate forms of immunotherapy such as vaccines and cellular therapies that are also historically known to exclude PLWH, however we chose to restrict the scope of this study due to the emerging evidence specifically studying ICI use in PLWH for safety and efficacy. Many trials in this study also combined multiple other study drugs, procedures, or radiation therapy with ICIs. This introduces the potential confounding variable that PLWH were excluded not due to ICI use but due to another study element.

In summary, PLWH have historically and continue to be excluded from most cancer ICI trials, limiting this at-risk population’s access to potentially beneficial therapies despite revolutionary breakthroughs in HIV treatments. Adherence to the FDA 2020 guidance for PLWH inclusion in cancer trials would expand therapeutic options for PLWH while the effects of ICIs in PLWH with inadequate immune function are further studied to develop evidence-based clinical decisions for trial inclusion. Overall, cancer ICI clinical trial norms must continue to prioritize patient safety but allow for further inclusion of PLWH to ensure equitable health care access.

## Methods

### Trial cohort collection

To determine the number of cancer ICI clinical trials excluding and conditionally including PLWH, two cohorts of trials from the ClinicalTrials.gov database were identified, one including trials with start dates between 1/1/2019 and 10/20/2020 (2019–2020 cohort) and one cohort with trial start dates between 1/1/2014 and 12/31/2014 (2014 cohort). Both cohorts of trials were assembled with the same methods by searching “cancer” for disease term and intervention terms “immunotherapy, PD-1, PD-L1, CTLA-4, pembrolizumab, nivolumab, atezolizumab, durvalumab, avelumab, cemiplimab, sintilimab, spartalizumab, ipilimumab, tremelimumab.” Non-therapeutic trials, trials enrolling multiple tumor types, and trials not treating with ICIs were excluded from analysis.

Since this study’s primary purpose was to evaluate the most recent rates of exclusion of PLWH in cancer ICI trials, the 2014 trial cohort was only used as a comparison to 2019–2020 to evaluate how the number of trials excluding PLWH has changed over time. Otherwise, all analyses were done using the 2019–2020 cohort.

### Trial categorization

In both the 2019–2020 and 2014 trial cohorts, all trials were categorized as “excluded,” “conditionally included,” or “included/not specified” based on review of trial eligibility criteria as listed on ClinicalTrials.gov. Exclusion criteria for each trial were reviewed for the terms HIV, AIDS, or acquired immune deficiency. Trials that allowed PLWH who met certain disease parameters (e.g. undetectable viral load, minimum CD4^+^ T-cell count) were categorized as “conditionally included.” For trials that did not mention PLWH in the trial ClinicalTrials.gov eligibility criteria, we checked the additional exclusion criteria listed in full study protocols on ClinicalTrials.gov, institutional trial databases, published papers, or posters. After this additional review, the remaining trials, which did not specifically exclude or conditionally include PLWH were categorized as “included/not specified.”

### Statistical analyses

The numbers of trials excluding PLWH were compared to the number of included/not specified trials using Fisher’s exact test to determine significance by tumor type, trial phase, and trial year. For sub-analysis by grouped tumor type, all trials were grouped into one of twelve subcategories: breast, cutaneous, gastrointestinal, genitourinary, gynecologic, head and neck, leukemia, lymphoma, multiple myeloma, nervous system, sarcoma and bone, or thoracic. Leukemia, lymphoma, and multiple myeloma trials were categorized as “hematologic malignancy” trials while the remainder were “solid tumor” trials. Phase II and earlier trials were considered “early phase” with “late phase” defined as phase II expanding into III or later trials. Five trials without listed trial phase on ClinicalTrials.gov were excluded from this analysis.

## Supplementary Information

Supplementary Information.

## References

[1] UNAIDS. *Global HIV & AIDS Statistics—2020 Fact Sheet*. https://www.unaids.org/en/resources/fact-sheet (UNAIDS, 2020).

[2] UNAIDS. *UNAIDS Urges Everyone to Make Some Noise for Zero Discrimination*. https://www.unaids.org/en/resources/presscentre/pressreleaseandstatementarchive/2017/february/20170301_zero-discrimination-day (UNAIDS, 2017).

[3] Global Network of People Living with HIV (GNP+). *The People Living with HIV Stigma Index*. https://www.stigmaindex.org/ (GNP+, 2020).

[4] Cook MR, Kim C (2019). Safety and efficacy of immune checkpoint inhibitory therapy in patients with HIV infection and advanced-stage cancer: A systemic review. JAMA Oncol..

[5] Grulich AE, van Leeuwen MT, Falster MO, Vajdic CM (2007). Incidence of cancers in people with HIV/AIDS compared with immunosuppressed transplant recipients: A meta-analysis. Lancet.

[6] Wang CC, Silverberg MJ, Abrams DI (2014). Non-AIDS-defining malignancies in the HIV-infected population. Curr. Infect. Dis..

[7] Silverberg MJ (2015). Cumulative incidence of cancer among persons with HIV in North America: A cohort study. Ann. Intern. Med..

[8] Coghill AE, Shiels MS, Suneja G, Engels EA (2015). Elevated cancer-specific mortality among HIV-infected patients in the United States. J. Clin. Oncol..

[9] Coghill AE, Pfeiffer RM, Shiels MS, Engels EA (2017). Excess mortality among HIV-infected individuals with cancer in the United States. Cancer Epidemiol. Biomark. Prev..

[10] Hernández-Ramírez RU, Shiels MS, Dubrow R, Engels EA (2017). Cancer risk in HIV-infected people in the USA from 1996 to 2012: A population-based, registry-linkage study. Lancet HIV.

[11] Yarchoan R, Uldrick TS (2018). HIV-associated cancers and related diseases. N. Engl. J. Med..

[12] Uldrick TS (2019). Assessment of the safety of pembrolizumab in patients with HIV and advanced cancer—A phase 1 study. JAMA Oncol..

[13] Ostios-Garcia L (2018). Safety and efficacy of PD-1 inhibitors among HIV-positive patients with non-small cell lung cancer. J. Thorac. Oncol..

[14] Gonzalez-Cao M (2020). Assessment of the feasibility and safety of durvalumab for treatment of solid tumors in patients with HIV-1 infection. JAMA Oncol..

[15] Food and Drug Administration. Cancer clinical trial eligibility criteria: Patients with HIV, hepatitis B virus, or hepatitis C virus infections. in *Federal Register* 41990–41992 (2020).

[16] American Society of Clinical Oncology, Friends of Cancer Research. *Cancer Clinical Trial Eligibility Criteria: Series of Four Draft Guidances*. https://www.asco.org/sites/new-www.asco.org/files/content-files/blog-release/pdf/ASCO-FOCR-Comments-FDA-Eligibility-Criteria_03May2019.pdf. Accessed 15 October 2020 (American Society of Clinical Oncology, 2019).

